# OpenSAFELY NHS Service Restoration Observatory 1: primary care clinical activity in England during the first wave of COVID-19

**DOI:** 10.3399/BJGP.2021.0380

**Published:** 2021-11-09

**Authors:** Helen J Curtis, Brian MacKenna, Richard Croker, Peter Inglesby, Alex J Walker, Jessica Morley, Amir Mehrkar, Caroline E Morton, Seb Bacon, George Hickman, Chris Bates, David Evans, Tom Ward, Jonathan Cockburn, Simon Davy, Krishnan Bhaskaran, Anna Schultze, Christopher T Rentsch, Elizabeth J Williamson, William J Hulme, Helen I McDonald, Laurie Tomlinson, Rohini Mathur, Henry Drysdale, Rosalind M Eggo, Kevin Wing, Angel YS Wong, Harriet Forbes, John Parry, Frank Hester, Sam Harper, Stephen JW Evans, Ian J Douglas, Liam Smeeth, Ben Goldacre, (The OpenSAFELY Collaborative)

**Affiliations:** The DataLab, Nuffield Department of Primary Care Health Sciences, University of Oxford, Oxford.; The DataLab, Nuffield Department of Primary Care Health Sciences, University of Oxford, Oxford.; The DataLab, Nuffield Department of Primary Care Health Sciences, University of Oxford, Oxford.; The DataLab, Nuffield Department of Primary Care Health Sciences, University of Oxford, Oxford.; The DataLab, Nuffield Department of Primary Care Health Sciences, University of Oxford, Oxford.; The DataLab, Nuffield Department of Primary Care Health Sciences, University of Oxford, Oxford.; The DataLab, Nuffield Department of Primary Care Health Sciences, University of Oxford, Oxford.; The DataLab, Nuffield Department of Primary Care Health Sciences, University of Oxford, Oxford.; The DataLab, Nuffield Department of Primary Care Health Sciences, University of Oxford, Oxford.; The DataLab, Nuffield Department of Primary Care Health Sciences, University of Oxford, Oxford.; London School of Hygiene and Tropical Medicine, London.; The DataLab, Nuffield Department of Primary Care Health Sciences, University of Oxford, Oxford.; The DataLab, Nuffield Department of Primary Care Health Sciences, University of Oxford, Oxford.; London School of Hygiene and Tropical Medicine, London.; The DataLab, Nuffield Department of Primary Care Health Sciences, University of Oxford, Oxford.; London School of Hygiene and Tropical Medicine, London.; London School of Hygiene and Tropical Medicine, London.; London School of Hygiene and Tropical Medicine, London.; London School of Hygiene and Tropical Medicine, London.; The DataLab, Nuffield Department of Primary Care Health Sciences, University of Oxford, Oxford.; London School of Hygiene and Tropical Medicine, London.; London School of Hygiene and Tropical Medicine, London.; London School of Hygiene and Tropical Medicine, London.; The DataLab, Nuffield Department of Primary Care Health Sciences, University of Oxford, Oxford.; London School of Hygiene and Tropical Medicine, London.; London School of Hygiene and Tropical Medicine, London.; London School of Hygiene and Tropical Medicine, London.; London School of Hygiene and Tropical Medicine, London.; London School of Hygiene and Tropical Medicine, London.; London School of Hygiene and Tropical Medicine, London.; TPP, Leeds.; London School of Hygiene and Tropical Medicine, London.; London School of Hygiene and Tropical Medicine, London.; London School of Hygiene and Tropical Medicine, London.; The DataLab, Nuffield Department of Primary Care Health Sciences, University of Oxford, Oxford.

**Keywords:** COVID-19, electronic health records, general practice, primary health care

## Abstract

**Background:**

The COVID-19 pandemic has disrupted healthcare activity. The NHS stopped non-urgent work in March 2020, later recommending services be restored to near-normal levels before winter where possible.

**Aim:**

To describe the volume and variation of coded clinical activity in general practice, taking respiratory disease and laboratory procedures as examples.

**Design and setting:**

Working on behalf of NHS England, a cohort study was conducted of 23.8 million patient records in general practice, *in situ* using OpenSAFELY.

**Method:**

Activity using Clinical Terms Version 3 codes and keyword searches from January 2019 to September 2020 are described.

**Results:**

Activity recorded in general practice declined during the pandemic, but largely recovered by September. There was a large drop in coded activity for laboratory tests, with broad recovery to pre-pandemic levels by September. One exception was the international normalised ratio test, with a smaller reduction (median tests per 1000 patients in 2020: February 8.0; April 6.2; September 6.9). The pattern of recording for respiratory symptoms was less affected, following an expected seasonal pattern and classified as ‘no change’. Respiratory infections exhibited a sustained drop, not returning to pre-pandemic levels by September. Asthma reviews experienced a small drop but recovered, whereas chronic obstructive pulmonary disease reviews remained below baseline.

**Conclusion:**

An open-source software framework was delivered to describe trends and variation in clinical activity across an unprecedented scale of primary care data. The COVD-19 pandemic led to a substantial change in healthcare activity. Most laboratory tests showed substantial reduction, largely recovering to near-normal levels by September, with some important tests less affected and recording of respiratory disease codes was mixed.

## INTRODUCTION

The ongoing pandemic caused by severe acute respiratory syndrome coronavirus 2 (SARS-CoV-2) has affected over 200 million people worldwide with at least 4.4 million deaths owing to COVID-19 as of August 2021.^1^ The need to direct resources towards patients requiring treatment for COVID-19 and to minimise opportunities for spread by reducing face-to-face contact between individuals meant that routine healthcare services faced significant levels of disruption. Shortly after declaring COVID-19 a public health emergency of international concern, the World Health Organization (WHO) reiterated their operational preparedness guidance. The intention of the guidance was to provide countries with advice on how to minimise direct and indirect mortality from COVID-19 through the continued provision of essential services. Recommendations included rapid assessment of healthcare capacity and the development of key performance metrics, and also highlighted the importance of keeping this data up to date.^2^^,^^3^ The NHS in England responded to the emerging pandemic by stopping non-urgent work in hospitals, and suggesting that, where possible, patients should have non-urgent primary care appointments remotely.^4^

In a rapid assessment conducted in May 2020, WHO found that across the world there has been a considerable impact on the treatment of people with non-communicable disease (NCDs, non-infectious diseases not passed from person to person) caused by severe disruption to the delivery of national healthcare services.^5^ Subsequently, NHS England issued guidance on the ‘third phase’ of the NHS response to COVID-19 on 21 July 2020. One of the many recommendations was to restore NHS services to near-normal levels where clinically appropriate before winter, while remaining vigilant for a second wave.^6^

OpenSAFELY is a new secure analytics platform for electronic patient records built by the author group on behalf of NHS England to deliver urgent academic and operational research during the pandemic:^7^ analyses can currently run across all patients’ full raw pseudonymised primary care records at 40% of English general practices, with patient-level linkage to various sources of secondary care data; all code and analysis is shared openly for inspection and re-use. A stated aim of OpenSAFELY is to assess ‘COVID aftershocks’ where data are monitored to measure and mitigate the indirect health impact of COVID-19.^8^ In order to produce the best possible insights across a range of diverse topics using this huge volume of activity and data, this author group is working with NHS England to create a programme of work that is called the OpenSAFELY NHS Service Restoration Observatory.

**Table:** How this fits in

During the COVID-19 pandemic, routine healthcare services in England faced significant levels of disruption, and NHS England recommended restoring NHS services to near-normal levels before the winter of 2020. This study found that, compared with activity in 2019, many pathology tests and much respiratory activity in primary care saw significant activity reductions from April to September 2020, largely recovering to near-normal levels by September, and some important tests were maintained at near-normal levels throughout. The authors are further developing the OpenSAFELY NHS Service Restoration Observatory for real-time monitoring and feedback for frontline clinicians and managers, to help measure and mitigate the ongoing indirect impact of COVID-19 on health and the NHS.

Traditionally researchers using electronic health records (EHRs) data create bespoke manually curated ‘codelists’ to identify certain diseases or units of healthcare activity. However, as the scale of raw data is unprecedented, a data-driven approach is initially being deployed utilising natural hierarchies contained within Clinical Terms Version 3 (CTV3) code structures (Box 1). The insights generated will then be manually reviewed and prioritised by groups of clinicians and commissioners for further analysis. The aim is to rapidly identify all important changes in clinical practice that have been collaboratively determined by clinicians and commissioners to be of high clinical importance, and then prioritise each relevant activity change for either remedial activity, additional monitoring, feedback to practices and regions, or further exploration.

**Box 1.** Clinical Terms Version 3 (CTV3) codes

CTV3 is a comprehensive computerised coding dictionary used by clinicians to record key clinical information about patients and also associated tests, diagnosis, and medicines. CTV3 codes are used in the OpenSAFELY-TPP implementation, and fully align with the GP subset of SNOMED CT, the NHS standard.^9^ There are almost 300 000 codes in total, each five characters long. CTV3 codes can be organised into hierarchies much like a book with chapters. CTV3 has a tree data structure with ‘parent’ concepts describing broader clinical areas and ‘child’ codes of increasing specificity as you move down the hierarchy. For example, a concept such as ‘laboratory procedures’ will have ‘child’ codes that are more specific such as ‘haematology’, which will in turn be broken down further into increasingly detailed concepts. Most child codes can only have a single parent, although 3% have multiple parents.

In this article the first phase of this work is set out, describing trends and variation in clinical activity codes to evaluate NHS service restoration from the first wave of the pandemic in England. Two large clinical topic areas were pragmatically selected for this first phase of work: ‘respiratory disease’, because this encompasses infectious diseases and common NCDs such as asthma and chronic obstructive pulmonary disease (COPD); and ‘laboratory procedures’ including blood tests, because these are required in diagnosis and monitoring for a broad range of NCDs. A classification system was also developed to filter time trend data to identify changes of interest.

## METHOD

### Study design

General practice clinical activity was described by conducting a retrospective cohort study using raw data from English NHS general practices.

### Data source

Primary care records managed by TPP were analysed through OpenSAFELY, a data analytics platform created by the author team on behalf of NHS England to address urgent COVID-19 research questions (https://opensafely.org). OpenSAFELY provides a secure software interface allowing researchers to run statistical analysis code across pseudonymised primary care patient records from England in near real time within the EHR vendor’s highly secure data centre, avoiding the need for large volumes of potentially disclosive pseudonymised patient data to be transferred off-site. This, in addition to other technical and organisational controls, minimises any risk of re-identification. Pseudonymised datasets from other data providers are securely provided to the EHR vendor and linked to the primary care data.

The dataset contains information on 23.8 million people registered with GP surgeries using TPP SystmOne software at 30 September 2020. It includes pseudonymised data such as coded diagnoses, medications, and physiological parameters; no free-text data are included. Practices are identified by pseudonymised codes only. Further details on information governance can be found in Supplementary Appendix S1.

### Study population

All patients registered with any practice using TPP EHR software (those who were registered as of 30 September 2020) were included. All coded events between January 2019 and the end of September 2020 for this cohort were included. Coded events cover clinical diagnoses, symptoms, observations, investigations, administrative activities, and other information recorded about patients. Codes may be manually entered by GPs/nurses or other practice staff, generated automatically when certain activities are carried out such as completing forms or templates, or derived from external sources such as secondary care.

### Data processing

Data was grouped at the practice level. Each patient’s latest practice (as at 30 September 2020) was used as their assigned practice for all activity throughout the study period. A data-driven approach was employed to capture the most common coded events in primary care. Codes were ranked according to the number of total occurrences in January to September 2020, excluding those codes with <1000 occurrences. The total population in each practice was calculated as the total registered patients at 30 September 2020 and the same value used for every month.

### Clinical code classification

EHR systems in UK primary care have historically used a number of different clinical terminologies, including Read codes Version 2 (V2) and the CTV3 (Box 1). Following the issue of a recent NHS standard, all primary care systems must now be compliant with SNOMED CT.^9^ More specifically, GP systems use a specific reference subset of the whole SNOMED CT terminology, known as the ‘GP subset’. This subset very closely mirrors CTV3, the terminology historically used by TPP systems. There is a comprehensive, accurate mapping table between CTV3 and the UK ‘GP subset’ of SNOMED CT. Users of TPP GP EHR software default to work in SNOMED CT, but can choose to view records and browse codes using CTV3, to facilitate their transition to the new terminology. For the OpenSAFELY-TPP analysis here, the authors have worked within the CTV3 framework, to work rapidly and utilise its hierarchy structures.

CTV3 codes often give great detail on specific clinical findings or diagnoses. The authors of this current study therefore classified codes into a number of groups to ascertain more general trends. This was achieved in two ways. First, CTV3 supports a comprehensive parent–child concept hierarchy, with defined relationships between different clinical concepts (Box 1). The authors have utilised this hierarchy to support the classification of codes into high-level groups. Second, these groups were supplemented using the broad historical ‘category’ structure of older terminologies, where parent–child relationships can be ascertained by a common set of initial characters. The authors acknowledge that this second approach can produce a small number of inappropriate or missing codes. It has, however, made it possible to work very rapidly to identify general trends. This is discussed further in the ‘Strengths and limitations’ section of this article.

As an example, individual CTV3 codes were grouped into high-level topics using their first two digits, for example, 42, ‘haematology’. Second, codes were grouped by their first three digits to give a further breakdown of activities (for example, 42Q, ‘blood coagulation test’). Although this approach generally produces logical groupings, there may be some examples where this is not the case, for example, ‘asthma’ (H33...) is grouped under ‘chronic obstructive lung disease’ (H3...), now considered an outdated classification. Further, these groups are non-exhaustive, because some codes related to these topics do not fall under this natural hierarchy; these ungrouped CTV3 codes (most commonly beginning with ‘X’ or ‘Y’) are therefore presented individually.

### Code selection

Codes and groups were mapped to high-level ‘concepts’ (Box 1) to assist with categorising them into related topics. Codes were selected for each topic using concepts and/or keyword searches of code descriptions. For each topic up to 75 of the most commonly occurring codes were selected.

- *Pathology*: codes were identified under the following concepts: ‘laboratory procedures’ (‘4....’), ‘laboratory test’ (‘X7A0B’), and ‘laboratory test observations’ (‘X76sW’).
- *Respiratory*: codes were identified based on the keywords ‘respiratory’, ‘asthma’, ‘COPD’, ‘chronic obstructive’, and ‘inhaler’. Keywords related to COVID-19 were not specifically included, so it is likely that some but not all COVID-19 codes will be captured.

### Missing data

Missing data may arise through lack of inclusion of patients who have died; but this is likely to only have a limited impact on most codes, except in those used for end-of-life issues that are relatively rare. Patients who have moved away from TPP practices were not included but those who moved into their latest practice during the study period were included. Certain codes may appear to have missing data owing to variable code use between practices, through, for example, selection of valid alternatives, use of different tools or templates, differing practice administrative processes, or use of on-screen tools for which codes do not get recorded. The authors could not account for these but expect that they will be generally consistent within each practice over time.

### Study measures

The monthly incidence of each code (or group of codes) per 1000 registered patients at each practice was calculated. The median and deciles across all practices each month for each code were calculated, after excluding practices that never used that code in the study period. Time trend charts are used to present data. All charts were collaboratively reviewed by clinicians and researchers; selected charts are shown in the ‘Results’ section to illustrate key patterns, with additional selected charts in Supplementary Appendix S2, and all charts are provided in the associated OpenSAFELY GitHub repository.^10^

In the case of all grouped (‘parent’) codes, a table of the top (up to) five ‘child’ codes is presented to illustrate examples of individual codes captured, and time trends of the top most common code have been plotted. Fewer than five child codes being shown indicates that fewer than five codes exist in the group, or fewer than five reached the 1000 minimum 2020 activity threshold.

### Classification of service restoration

With each chart the median value and interdecile range (IDR) for February, April, and September 2020 are displayed. February was selected as the last full month before lockdown measures were instigated in March, whereas April was identified as the first full month after full lockdown. September was the latest full month at the time of initiating this analysis and before the ‘second wave’ of infections would have been expected to influence health services once again. To aid interpretation, an approximate classification based on changes to the median compared with the same month the previous year is provided, which is defined as the study ‘baseline’ (Box 2).

**Box 2.** Service change classification

For April and September: *no change*: activity remained within 15% of the baseline level; *increase*: an increase of >15% from baseline; *small drop*: a reduction of between 15% and 60% from baseline; *large drop*: a reduction of >60% from baseline. Overall classification: *no change*: no change in both April *and* September; *increase*: an increase in either April *or* September; *sustained drop*: a small or large drop in April, which has *not* returned to within 15% of baseline by September 2020; *recovery*: a small or large drop in April, which returned to within 15% of baseline (‘no change’) by September 2020.

### Software and reproducibility

Data management was performed using Python and the OpenSAFELY software, with data extracted via SQL Server Management Studio and analysis carried out using Python. All of the code used for data management and analyses is openly shared online for review and re-use (https://github.com/opensafely/restoration-observatory-intro-notebook).

### Patient and public involvement

This analysis relies on the use of large volumes of patient data. Ensuring patient, professional, and public trust is therefore of critical importance. Maintaining trust requires being transparent about the way OpenSAFELY works, and ensuring patient voices are represented in the design of research, analysis of the findings, and considering the implications. For transparency purposes a public website has been developed that provides a detailed description of the platform in language suitable for a lay audience; the authors will be co-developing an explainer video; and have presented at a number of online public engagement events to key communities. To ensure the patient voice is represented, the authors are working closely with appropriate medical research charities. In this instance, the draft paper has been shared with the Association of Medical Research Charities for general comment via a webinar and online feedback form, and specifically to Asthma UK and the British Lung Foundation for feedback from the perspective of those most affected by these findings, before publication in a peer-reviewed journal.

## RESULTS

This study included 23 878 341 registered patients across 2546 practices. Between 38.8% and 100% of practices were represented in each chart. From January to September 2020 for laboratory procedures 83.6 million recorded events grouped under two-digit ‘parent’ codes were identified, and 235.7 million events grouped at three digits where possible, or at the full code level. By comparison, for respiratory disease 1.4 million events at the two-digit parent code level were identified, and 22.3 million events grouped at three digits where possible, or at the full code level. All charts are shown in the associated OpenSAFELY GitHub repository, with selected highlights shown in the figures and in Supplementary Appendix S2.

### Pathology

**Figure 1.**
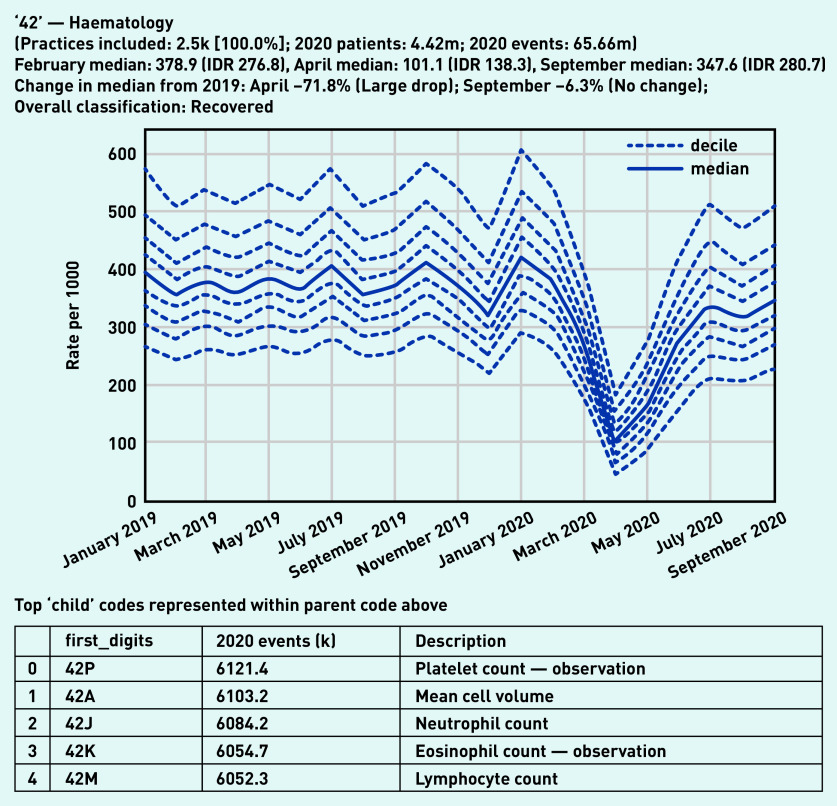
*Recording of codes grouped under ‘haematology’ across TPP practices in England (January 2019 to September 2020). The group includes CTV3 codes that begin with ‘42’ and is not necessarily an exhaustive collection of every activity related to haematology. The top five codes represented within this group are listed under the graph.* *CTV3 = Clinical Terms Version 3. IDR = interdecile range. k = thousand. m = million.*

Most two-digit high-level codes showed a similar pattern — a large drop (Box 2) in activity in April 2020, recovering to normal levels over the summer. A representative example, haematology laboratory tests activity, is shown in Figure 1. The variation observed between practices in September (IDR 280.7) was similar to before the pandemic in February (IDR 276.8). More broadly, many individual tests had similar large drops in activity in April followed by substantial recovery, such as liver function, serum alkaline phosphate, serum creatinine, and serum potassium (see Supplementary Appendix S2).

**Figure 2.**
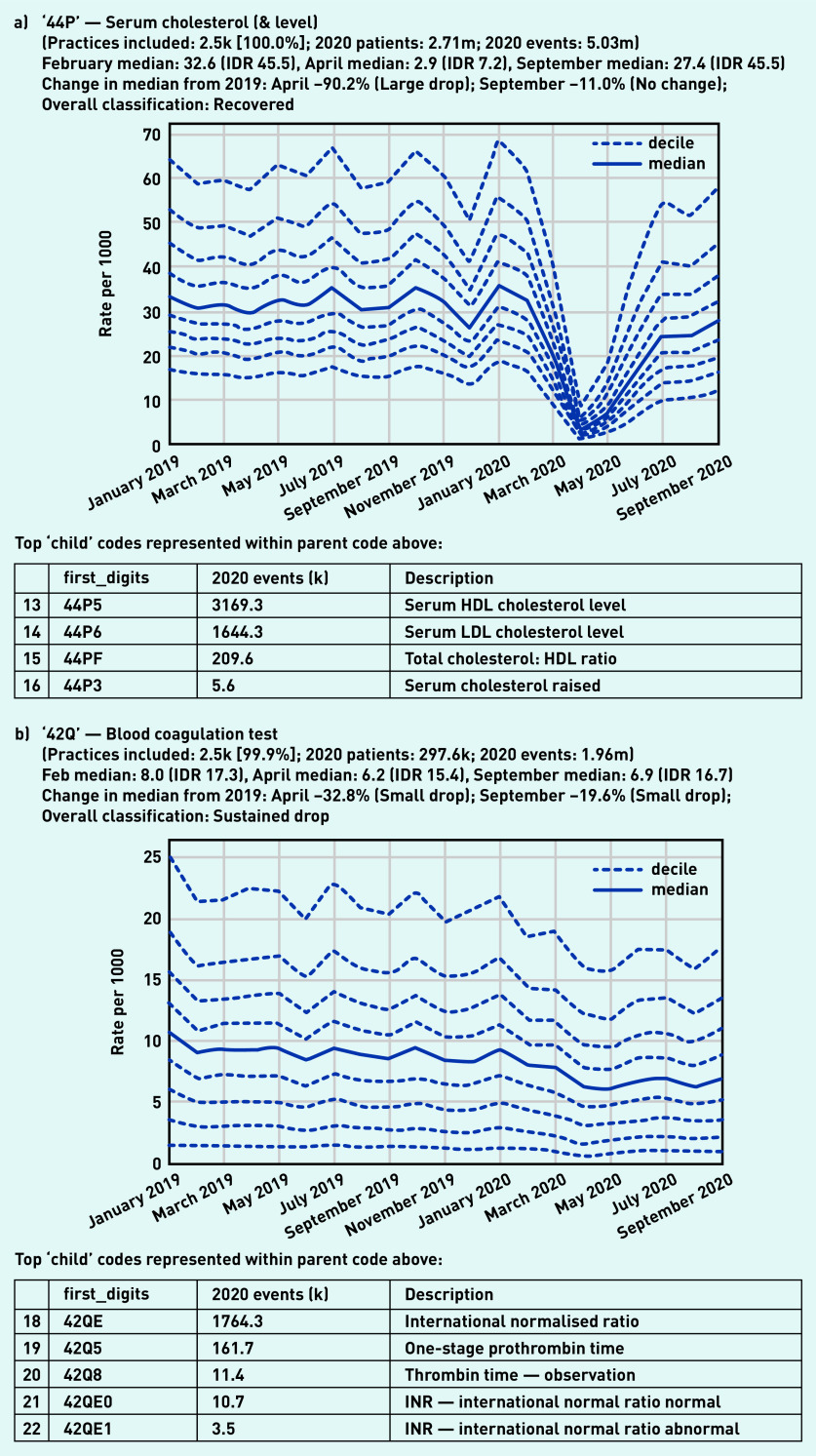
*Recording of grouped subsets of pathology codes across TPP practices in England (January 2019 to September 2020). a) ‘Serum cholesterol (& level)’. The group includes CTV3 codes that begin with ‘44P’ and is not necessarily an exhaustive collection of every activity related to serum cholesterol testing. b) ‘Blood coagulation test’. The group includes CTV3 codes that begin with ‘42Q’ and is not necessarily an exhaustive collection of every activity related to blood coagulation testing. The top five codes represented within this group are listed under the graph. CTV3 = Clinical Terms Version 3. HDL = high-density lipoprotein.* *IDR = interdecile range. INR = international normalised ratio. k = thousand. LDL = low-density lipoprotein. m = million.*

The largest drop was observed in ‘serum cholesterol (& level)’ (Figure 2a) with the median falling by 90.2% from the previous year to 2.9 tests per 1000 in April. This recovered to near-normal levels by September, with the variation between practices similar to that seen in February (IDR 45.5 in both months). In contrast, blood coagulation tests such as the international normalised ratio (INR), used to support management of anticoagulation, showed only a small drop in April (median 6.2/1000) compared with the pre-pandemic level (median 8.0/1000; Figure 2b).

### Respiratory disease

The codes under respiratory disease can be broadly divided into those relating to symptoms and acute infections, and those relating to long-term conditions.

#### Symptoms and acute infections

**Figure 3.**
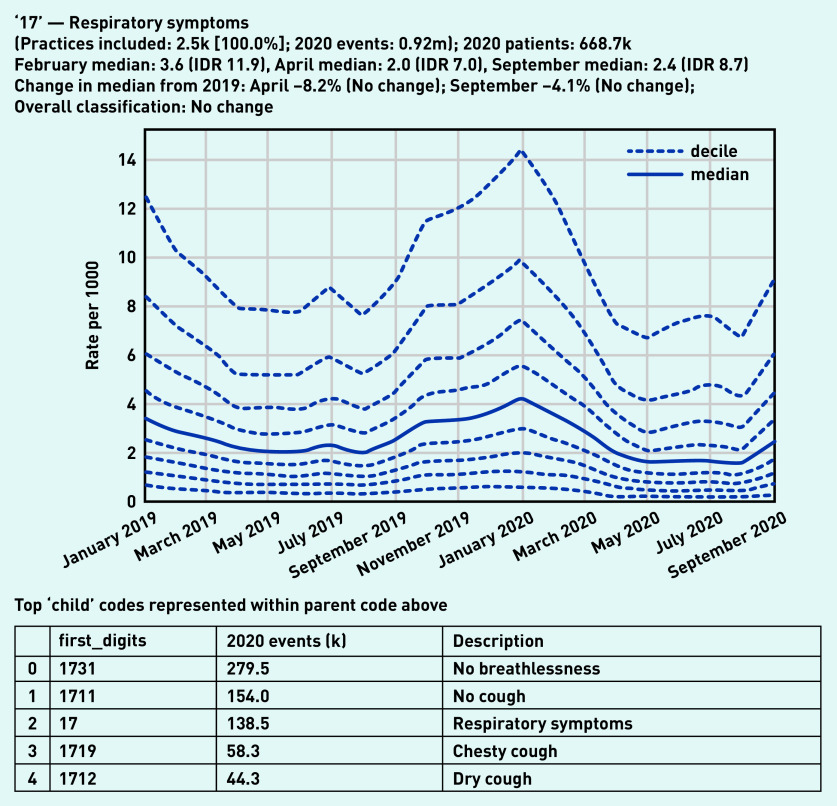
*Recording of codes grouped under ‘respiratory symptoms’ across TPP practices in England (January 2019 to September 2020). The group includes CTV3 codes that begin with ‘17’ and is not necessarily an exhaustive collection of every activity related to respiratory symptoms. The top five codes represented within this group are listed under the graph.* *CTV3 = Clinical Terms Version 3. IDR = interdecile range. k = thousand. m = million.*

**Figure 4.**
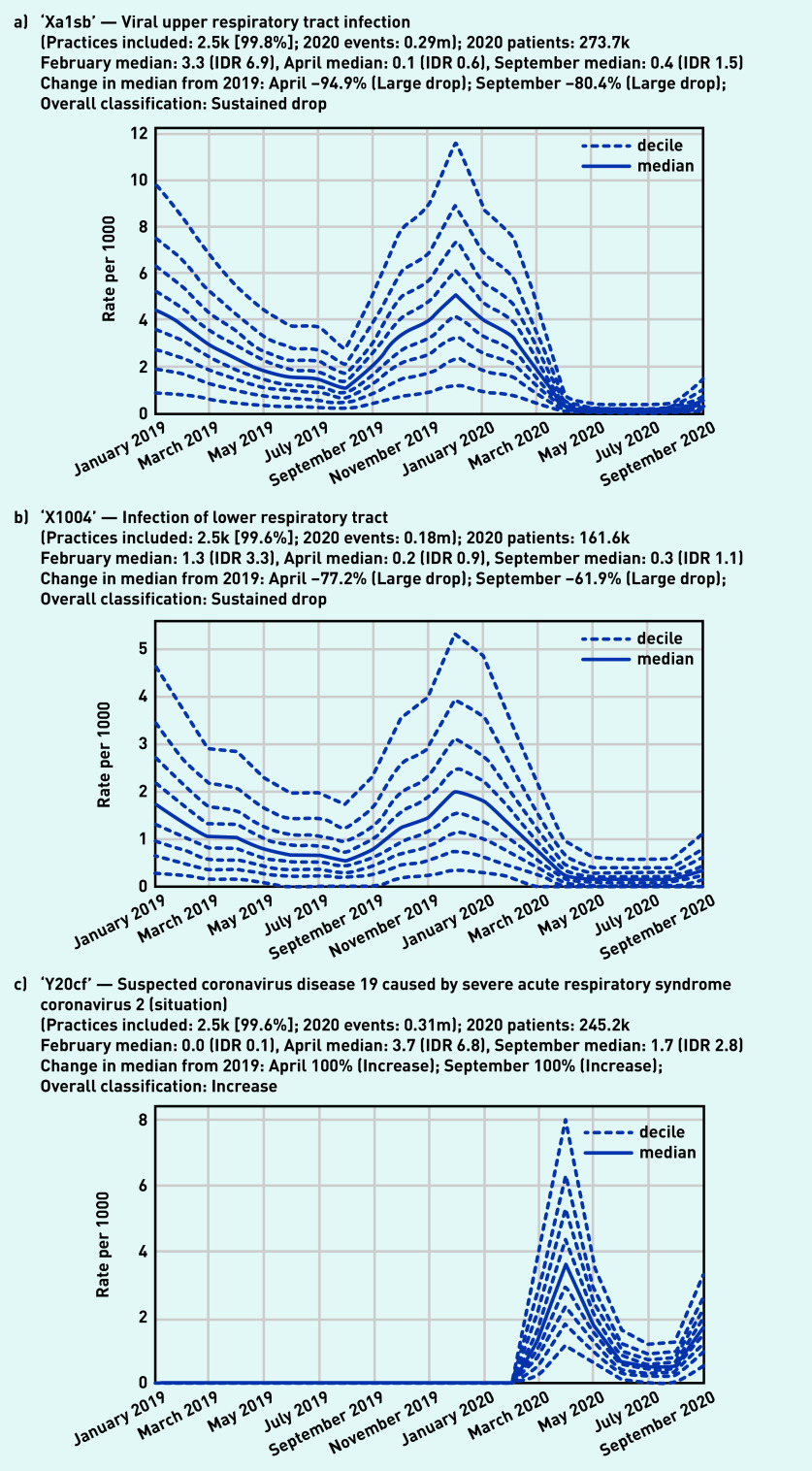
*Recording of selected individual codes related to respiratory infections across TPP practices in England (January 2019 to September 2020). a) Viral upper respiratory tract infection, b) infection of lower respiratory tract, and c) suspected COVID-19. These were the most common codes identified for these activities, but other codes may also be used to record the same or similar activities.* *DR = interdecile range. k = thousand. m = million.*

Recording of codes grouped under ‘respiratory symptoms’ remained relatively stable throughout the observed period (Figure 3), largely following an expected seasonal pattern. Three individual codes were selected that demonstrate broad patterns in the recording of acute respiratory infections (Figure 4). ‘Viral upper respiratory tract infection’ (‘Xa1sb’) exhibited a large and sustained drop compared with pre-pandemic levels, remaining substantially lower than in the previous year (September –80.4%, Figure 4a). Similarly, ‘infection of lower respiratory tract’ (‘X1004’) experienced a large drop (April –77.2%, Figure 4b). As the recording of other respiratory infections began to drop, ‘Suspected coronavirus disease 19 caused by severe acute respiratory syndrome coronavirus 2’ (‘Y20cf’) increased rapidly during spring (April median 3.7/1000, IDR 6.8, Figure 4c; other codes related to COVID-19 can be found in Supplementary Appendix S2). This then decreased dramatically over the summer before increasing again in September (median 1.7/1000, IDR 2.8).

#### Long-term respiratory conditions

**Figure 5.**
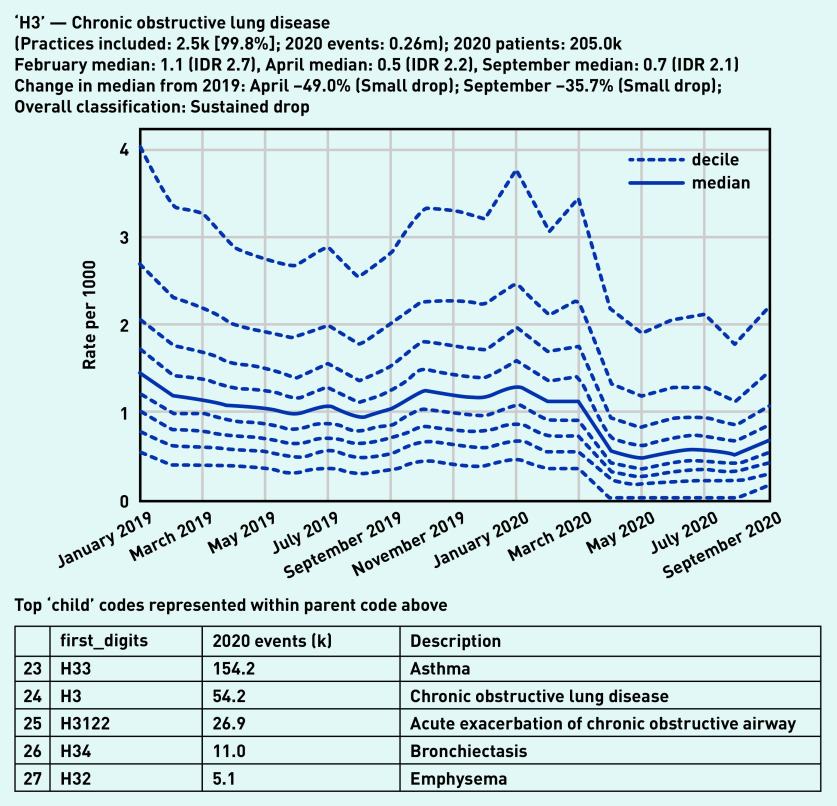
*Recording of codes grouped under ‘chronic obstructive lung disease’ across TPP practices in England (January 2019 to September 2020). The group includes CTV3 codes that begin with ‘H3’ and is not necessarily an exhaustive collection of every activity related to chronic obstructive lung disease. The top five codes represented within this group are listed under the graph.* *CTV3 = Clinical Terms Version 3. IDR = interdecile range. k = thousand. m = million.*

**Figure 6.**
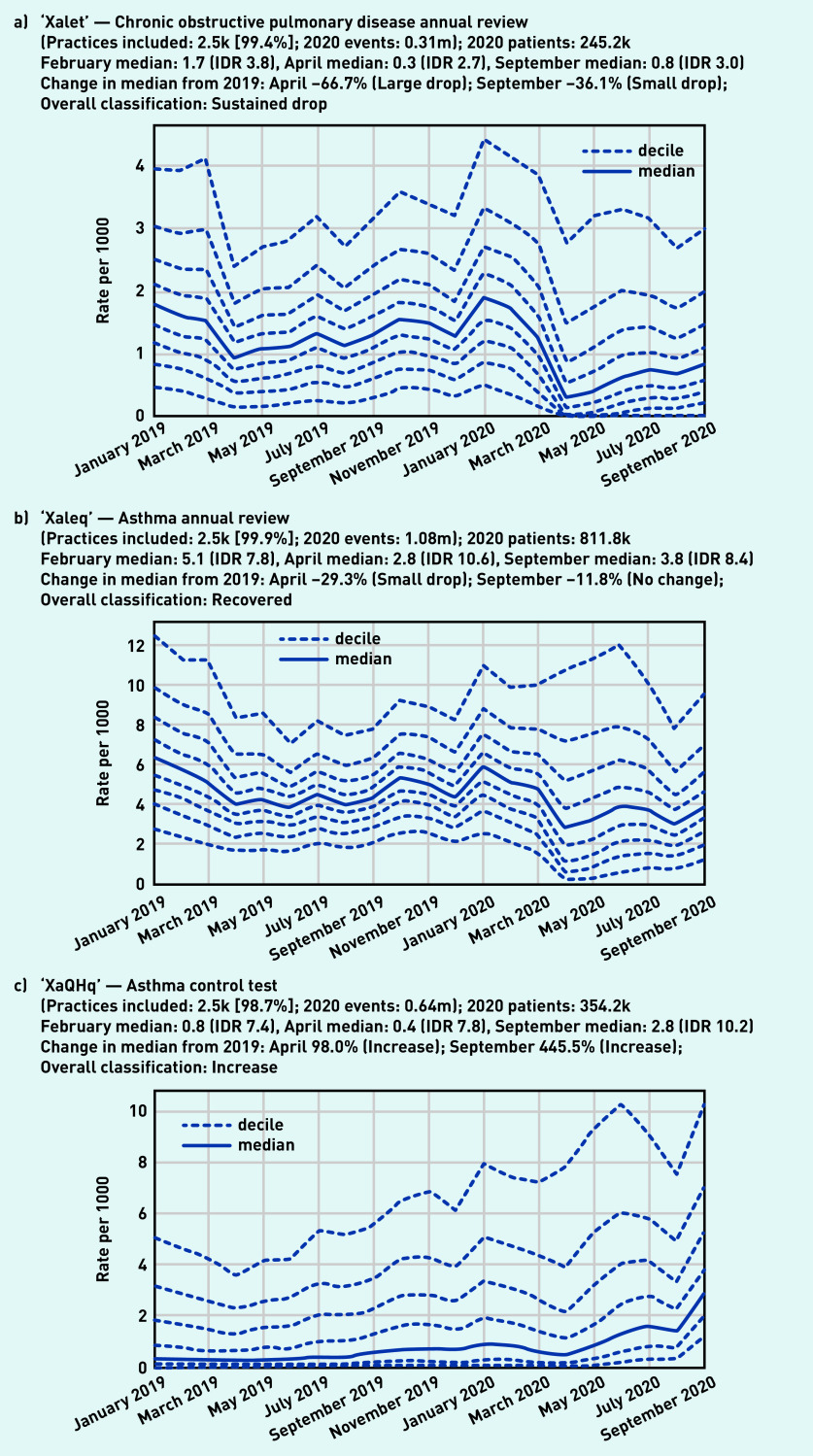
*Recording of selected individual codes related to annual reviews for long-term respiratory conditions across TPP practices in England (January 2019 to September 2020): a) chronic obstructive pulmonary disease annual review, b) asthma annual review, and (c) asthma control test. These were the most common codes identified for these activities, but other codes may also be used to record the same or similar activities.* *IDR = interdecile range. k = thousand. m = million.*

Codes for both ‘asthma’ and ‘chronic obstructive lung disease’ were included within the high-level group of codes beginning ‘H3’, ‘chronic obstructive lung disease’, of which 260 000 codes were recorded in 2020 by 99.8% (*n* = 2500) of practices (Figure 5). Overall, this group showed a small drop in April (median 0.5/1000, –49.0%) with some recovery by September (median 0.7/1000, –35.7% compared with the previous September). There were similar trends in some individual codes related to monitoring of these conditions, such as ‘chronic obstructive pulmonary disease annual review’ (‘Xalet’, Figure 6a), with a large drop followed by partial recovery (April –66.7%, September –36.1%). Following a broadly similar pattern, ‘asthma annual review’ (‘Xaleq’, Figure 6b) exhibited a smaller drop (April median 2.8/1000, –29.3% from the previous April) but with widening variation (IDR 10.6, compared with 7.8 in February with median 5.1/1000). This code had a more complete recovery, to a median of 3.8 in September (–11.8% from the previous September), but with wide variation persisting (IDR 8.4). Several other codes related to asthma monitoring had a gradually increasing pattern, with a dramatic increase in September, including ‘asthma control test‘ (‘XaQHq’, Figure 6c), ‘number of asthma exacerbations in past year’ (‘XaINh’; Supplementary Appendix S2), and ‘asthma self-management plan review’ (‘XaYZB’; Supplementary Appendix S2). ‘Asthma control test’, previously a relatively unusual code but with high variation (February median 0.8/1000, IDR 7.4), rose sharply in September to a median of 2.8/1000 (IDR 10.2, Figure 6c).

## DISCUSSION

### Summary

It was possible to successfully generate data on trends and variation in clinical activity across the records of 40% of all practices in England. Substantial and widespread changes in the pattern of clinical activity for laboratory procedures and respiratory disease in primary care since the onset of the COVID-19 pandemic were identified. Broadly, clinical activity related to laboratory procedures such as blood tests declined substantially after ‘lockdown’ but recovered quickly over the summer. Blood tests to manage high-risk anticoagulants, however, were prioritised and did not experience a similar drop off in activity. Recording of respiratory symptoms overall, including cough and breathlessness, remained relatively constant, although codes related to viral respiratory illness substantially declined compared with previous activity, with the exception of those specifically related to COVID-19. A small decline was observed associated with high-level codes for long-term respiratory conditions such as COPD and asthma. Activity related to asthma annual reviews experienced a small drop but has since recovered, whereas COPD annual reviews are still below baseline.

### Strengths and limitations

The key strengths of this study are the scale and completeness of the underlying raw EHR data. The OpenSAFELY platform runs analyses across an unprecedented scale of data — the full dataset of all raw, single-event-level clinical events for all patients at 40% of all GP practices in England, including all tests, treatments, diagnostic events, and other salient clinical and demographic information for 23.8 million patients. By contrast the Clinical Practice Research Datalink (CPRD) dataset contains records for a substantially smaller number of current patients spread across two databases; and the General Practice Extraction Service dataset held by NHS Digital contains a much smaller amount of data on each individual patient. OpenSAFELY also provides data in near real time, providing unprecedented opportunities for audit and feedback to rapidly identify and resolve concerns around health service activity — the delay from occurrence of a clinical event to it appearing in the OpenSAFELY platform varies from 2–9 days. This is substantially faster than any other source of GP data, including those giving much less complete records. It is also faster than any source of large-scale secondary care data as the Secondary Users Service dataset, containing duration and main activity for each hospital event, is coded several weeks after completion (not commencement) of the clinical episode or spell.

Some limitations are also recognised. A data-driven approach was used, capitalising on the historical CTV3 hierarchy where possible, in combination with the CTV3 concept hierarchy. There may be other codes relevant to laboratory procedures and respiratory conditions that have been omitted; the authors intend to iterate the approach used with manual curation of ‘codelists’ and utilisation of SNOMED CT hierarchies to better understand activity.

With the exception of a small amount of legally restricted data, all occurrences of codes are included, and they do not necessarily indicate unique or new events, for example, one patient encounter could generate several similar codes, one patient might have similar diagnoses recorded on multiple occasions over time, or practices might bulk-import information. Coding activity was studied and some apparent changes may represent changes in coding behaviour. The large population covered here is likely to be broadly representative of the whole of England’s population, but some coding practices may vary between different EHR systems, so not all of these findings will be generalisable.

Finally, codes were counted against patients, who were then allocated to their latest registered practice as at the end of the study period. All patients with an active registration at the end of the study were included, so past activity for patients who registered during the study period was included under their latest practice, even where it occurred in a non-TPP practice. A very small number of patients may have overlapping registrations, meaning any activity they had will be counted against multiple practices. Patients who died or de-registered from TPP practices throughout the study period were not included. Overall, activity counts were up to 6%–8% lower than database totals in the earliest months of the study period.

### Comparison with existing literature

A recent systematic review of healthcare usage during the pandemic, encompassing 81 studies across 20 countries, found that healthcare utilisation reduced by approximately one-third during the pandemic.^11^ This is in line with the findings in the current study of substantial reductions in April and May. However, by using near real-time data it was also possible to detect ongoing recovery. WHO also found significant disruption to countries’ healthcare capacity for NCDs in May^5^ and highlighted the importance that countries ‘build back better’ healthcare services for people with NCD, partially as they are more likely to experience adverse outcomes from COVID-19.^7^

Some studies have been conducted in the UK CPRD that covers 13% of the UK population. One such study found a rate reduction of 0.77 for haemoglobin A1c testing among people with type 2 diabetes in England in March 2020 and a similar reduction in new type 2 diabetes diagnoses compared with 10-year averages;^12^ there was some recovery in the following months, but not reaching the normal range until December 2020. Another CPRD study found substantial reductions in primary care contacts for acute physical and mental conditions such as depression, self-harm, diabetic emergencies, and COPD/asthma exacerbations, with ‘limited recovery’ by July 2020;^13^ similarly this current study found recovery had occurred in a broad range of coded activity by September. In a separate article this current author group found there was no drop-off in INR activity after adjustment for the amount of people on warfarin, the medicine that requires routine INR monitoring.^14^ Some national data sources can also be used to assess trends in NHS activity, such as cancer referral and treatment^15^ and primary care prescribing.^16^ However, such high-level datasets are useful for giving an overview of activity (and, in the case of prescribing data, regional/local breakdowns), but do not permit linkage to demographic or clinical features as is possible with patient-level records.

### Implications for research and practice

To the authors’ knowledge this study is both the first operational research output of its scale using NHS GP data and the first to take a primarily data-driven approach to understanding changes in general practice. Two broad areas for further research are proposed. The first area, building on this approach, is a programme of research describing the changes in healthcare activities across a broad range of clinical areas. Second, to support WHO and NHS England recommendations to ‘build back better’, it is necessary to understand the causes of the changes observed, for example, determining genuine changes in disease prevalence or presentation, versus changes in delivery of healthcare services, or changes in coding behaviour. As an immediate first step the authors have established a clinical advisory group comprising GPs, pharmacists, relevant specialists, and national clinical advisers with patient and public involvement being coordinated by the Association of Medical Research Charities. This group will review similar data to those published in this report on a diverse range of subject areas such as mental health, cardiovascular disease, and diabetes. The authors will synthesise feedback and openly share short written reports describing the findings, identifying any important signals, actionable insights that are suitable for interactive dashboard candidates, and research recommendations. The authors will then actively seek community feedback to iterate these reports. Ultimately, working closely with EHR providers, the aim is to present actionable data insights directly back to individual practices to improve patient care and inform response to COVID-19.

The COVID-19 pandemic has brought new challenges for the NHS to deliver safe and effective routine care. This study has shown sharp changes in delivery of activity related to clinical care with quick recovery observed in certain activities. Although some important blood tests remained relatively stable throughout the period, most pathology test activity experienced substantial reductions, largely recovering to near-normal levels by September. This may be an indicator of an effective general practice system independently responding in the midst of a global health emergency to deprioritise inessential tests at the height of the pandemic and quickly recovering as the ‘first wave’ subsided. The proposed NHS Service Restoration Observatory can support evaluation of national policies around service restoration and additionally provide opportunities for near real-time audit and feedback to rapidly identify and resolve concerns around health service activity. In particular, the hope is that data tools such as this one can be used to ensure continuity of high-priority clinical services during subsequent waves of the pandemic.

In conclusion, substantial changes in activity from April to September 2020 in healthcare service delivery as a result of the COVID-19 pandemic were observed. Although some important tests remained relatively stable throughout the period, most pathology test activity experienced substantial reductions, largely recovering to near-normal levels by September. Records of respiratory infections decreased with the exception of codes related to COVID-19, whereas activity for other respiratory disease codes was mixed. The authors are now further developing the OpenSAFELY NHS Service Restoration Observatory for real-time monitoring and feedback, using primary care data to measure and rapidly mitigate the indirect health impact of COVID-19 on the NHS.

## Data

Access to the underlying identifiable and potentially re-identifiable pseudonymised electronic health record data is tightly governed by various legislative and regulatory frameworks, and restricted by best practice. The data in OpenSAFELY is drawn from General Practice data across England where TPP is the Data Processor. TPP developers (Chris Bates, Jonathan Cockburn, John Parry, Frank Hester, and Sam Harper) initiate an automated process to create pseudonymised records in the core OpenSAFELY database, which are copies of key structured data tables in the identifiable records. These are linked onto key external data resources that have also been pseudonymised via SHA-512 one-way hashing of NHS numbers using a shared salt. DataLab developers and PIs (Ben Goldacre, Liam Smeeth, Caroline E Morton, Seb Bacon, Alex J Walker, William Hulme, Helen J Curtis, David Evans, Peter Inglesby, Simon Davy, George Hickman, Krishnan Bhaskaran and Christopher T Rentsch) holding contracts with NHS England have access to the OpenSAFELY pseudonymised data tables as needed to develop the OpenSAFELY tools. These tools in turn enable researchers with OpenSAFELY Data Access Agreements to write and execute code for data management and data analysis without direct access to the underlying raw pseudonymised patient data, and to review the outputs of this code. All code for the full data management pipeline — from raw data to completed results for this analysis — and for the OpenSAFELY platform as a whole is available for review at github.com/OpenSAFELY.
